# Comparative efficacy and safety of CDK4/6 inhibitors combined with endocrine therapies for HR+/HER2-breast cancer: Systematic review and network meta-analysis

**DOI:** 10.1016/j.heliyon.2024.e31583

**Published:** 2024-05-21

**Authors:** Fei Tong, Yi Lu, Hong-Fang Ma, Jun Shen

**Affiliations:** a Department of General Surgery, The People's Hospital of Longyou County, Quzhou, Zhejiang Province, China; b Department of Surgical Oncology, Sir Run Run Shaw Hospital, Zhejiang University School of Medicine, Hangzhou, Zhejiang Province, China; c Department of Plastic Surgery, Sir Run Run Shaw Hospital, Zhejiang University School of Medicine, Hangzhou, Zhejiang Province, China

**Keywords:** CDK4/6 inhibitors, Efficacy, Endocrine therapy, HR+/HER2-breast cancer, Meta-analysis, Safety

## Abstract

**Background:**

In recent years, the combination of targeted drugs, such as Cyclin-dependent kinase 4/6 (CDK4/6) inhibitors, with endocrine therapy (ET), has emerged as a new research focus in the treatment of hormone receptor-positive (HR+) human epidermal growth factor receptor 2 negative (HER2-) breast cancer. This network meta-analysis aimed to systematically evaluate the efficacy and safety of CDK4/6 inhibitors combined with ET for HR+/HER2-breast cancer.

**Methods:**

A systematic search was conducted across PubMed, Web of Science, Cochrane Library, and GeenMedical databases to identify randomized controlled trials investigating the use of CDK4/6 inhibitors in combination with endocrine therapy for the treatment of HR+/HER2-breast cancer. The search period spanned from the inception of each database up to February 29, 2024. Data analysis was conducted using Stata 14.0 and R 4.1.0 software.

**Results:**

A total of 20 randomized controlled trials (RCTs) were included in this study, investigating the effectiveness of four CDK4/6 inhibitors—Abemaciclib, Dalpiciclib, Ribociclib, and Palbociclib—when combined with ET for the treatment of HR+/HER2-breast cancer. The results indicated that Abemaciclib + ET, Dalpiciclib + ET, Palbociclib + ET, and Ribociclib + ET exhibited similar therapeutic effects in terms of improving objective response rate (ORR), disease control rate (DCR) and reducing the occurrence of fatigue, all of which were superior to ET alone. However, in terms of prolonging progression-free survival (PFS) and overall survival (OS), Dalpiciclib + ET significantly improved PFS compared to Ribociclib + ET, Palbociclib + ET, Abemaciclib and Palbociclib. Ribociclib + ET significantly improved OS compared to Palbociclib + ET. Regarding overall adverse reaction events (AREs), Dalpiciclib + ET had a higher incidence compared to Ribociclib + ET. The incidence of neutropenia caused by Dalpiciclib + ET was significantly higher compared to Palbociclib + ET, Ribociclib + ET, Abemaciclib, and Palbociclib. Abemaciclib + ET demonstrated the worst safety profile concerning diarrhea.

**Conclusion:**

Abemaciclib + ET likely represents the most effective option in terms of therapeutic effects, but it is prone to causing diarrhea and fatigue. On the other hand, Dalpiciclib + ET likely demonstrates the best efficacy in terms of PFS but exhibits the poorest safety profile, particularly in relation to neutropenia. Therefore, clinicians should exercise increased vigilance in monitoring and managing adverse effects when prescribing Abemaciclib + ET and Dalpiciclib + ET.

## 1 Introduction

Recent statistics have highlighted that breast cancer is the most commonly diagnosed cancer worldwide, with approximately 2.3 million new cases in 2020, and remains the leading cause of cancer death in women [1]. Within the spectrum subtypes of metastatic breast cancer, hormone receptor-positive (HR+)/human epidermal growth factor receptor 2-negative (HER2-) breast cancer emerges as a prevalent type with a poor prognosis [2]. The growth, proliferation, and metastasis of HR+/HER2-breast cancer cells are intricately regulated by the estrogen receptor signaling pathway [3], rendering endocrine therapy (ET) the cornerstone clinical intervention for hormone level regulation. Despite the effectiveness of ET in diminishing cancer recurrence and mortality rates among patients, it may trigger various adverse symptoms including neutropenia, diarrhea, fatigue, and menopausal symptoms [4]. In recent years, the confluence of targeted drugs such as Cyclin-Dependent Kinase4/6 (CDK4/6) inhibitors with ET has emerged as a burgeoning area of research. Numerous randomized controlled trials (RCTs) have conclusively demonstrated that the combination of CDK4/6 inhibitors with ET effectively manages HR+/HER2-breast cancer, curtails the dissemination of cancer cells, and markedly improves patients' quality of life and survival rates [[5], [6], [7], [8]].

CDK4/6 inhibitors combined with ET have become the standard of care for patients with HR+/HER2-metastatic breast cancer. Palbociclib [9], dalpiciclib [10], ribociclib [11], and abemaciclib [12] have all been approved by regulatory bodies such as the US Food and Drug Administration (FDA) and the European Medicines Agency. Sledge Jr et al. [13] found that Abemaciclib + ET (Fulvestrant) was superior to simple Fulvestrant in terms of progression-free survival (PFS) and objective response rate (ORR), but it was more prone to diarrhea and neutropenia, nausea and fatigue, and other adverse reactions events (AREs). Xu et al. [14] found that dalpiciclib plus fulvestrant significantly prolonged PFS versus placebo plus fulvestrant, but easily induced the most common grade 3 or 4 AREs, such as neutropenia and leukopenia. Iwata et al. found that Palbociclib + ET (Fulvestrant) can improve the PFS of HR+/HER2 breast cancer and is well-tolerated [15]. Im et al. found that Ribociclib + ET can improve the OS of HR+/HER2 breast cancer, and no new AREs were found [16]. Numerous clinical studies have demonstrated similar efficacy of the three CDK4/6 inhibitors in combination with ET, all of which significantly prolong the PFS and ORR of HR+/HER2-breast cancer patients [17]. However, direct comparisons between the three inhibitors combined with ET and a single CDK4/6 inhibitor or ET, as well as between the three inhibitors combined with ET, are still lacking, which hinders the selection of clinical drugs for HR+/HER2-breast cancer patients. This study aimed to use network meta-analysis to compare the efficacy and safety of CDK4/6 inhibitors combined with ET, providing a more evidence-based foundation for clinical drug selection in patients with HR+/HER2-breast cancer.

## 2 Materials and methods

### 2.1 Inclusion and exclusion criteria

Inclusion criteria: (1) study design: RCTs; (2) participants: patients diagnosed with HR+/Her2-breast cancer, with no restrictions on age, gender, disease subtype, or disease stage; (3) intervention measures: CDK4/6 inhibitors in combination with ET used in the experimental group, with no limitations on the dose or duration of treatment. The control group was treated with either CDK4/6 inhibitor or ET alone. Both groups underwent identical procedures and support measures, differing only in the intervention received; (4) outcomes: efficacy (ORR, DCR, OS, and PFS), AREs (overall ARE, neutropenia, diarrhea, and fatigue). Exclusion criteria: (1) duplicate literature; (2) literature where relevant outcomes could not be extracted; (3) literature containing errors; (4) literature with a limited number of included patients.

### 2.2 Literature search

A comprehensive systematic search was conducted across multiple databases including Pubmed, Embase, Cochrane Library, Web of Science, Scopus, and OVID, aiming to identify RCTs focusing on CDK4/6 inhibitors combined with ET in the treatment of HR+/HER2-breast cancer. The search period extended from the establishment of each database to March 10, 2022. Keywords utilized in the search strategy encompassed terms such as HR+/HER2-, breast cancer, endocrine therapy, and CDK4/6 inhibitor, as well as Abemaciclib, Ribociclib, Palbociclib, Trilaciclib, or Dalpiciclib. Further details regarding the search strategy can be referenced in Supplementary file 1.

### 2.3 Literature screening and data extraction

Following the “literature search” protocol, the collected literature underwent screening based on predefined "exclusion criteria". In cases where abstracts were inconclusive, full texts were reviewed to ascertain the number of included studies and corresponding ClinicalTrials.gov identifier. Literature containing redundant clinical data or studies with smaller sample sizes were excluded. Data extraction encompassed demographic details of study participants, disease characteristics, intervention protocols, and relevant outcome measures for both the experimental and control groups. These tasks were carried out by two researchers, with any discrepancies resolved through mutual discussion. If consensus couldn't be reached, a third researcher was consulted for resolution.

### 2.4 Risk of bias assessment

The “risk of bias” assessment tool, as outlined by Ref. [18], was employed to evaluate the methodological quality of the included studies. The assessment criteria included encompassed randomization, allocation concealment, blinding of subjects and evaluators, data integrity, selective reporting, and other potential biases. Each study was categorized as low risk, unclear risk, or high risk according to its research reports. Two researchers independently conducted evaluations and cross-checked the results. Any inconsistencies were deliberated upon, and a consensus was reached. In cases where consensus couldn't be achieved, a third researcher was consulted for a final decision.

### 2.5 Statistical analysis

Review manager 5.3 was utilized to generate the risk bias map, while the graph package of R 4.1.0 software was employed to construct the intervention network evidence map. Dichotomous variables were represented using odds ratio (OR). LnHR and selnHR were computed according to the hazard ratio (HR) and 95 % confidence interval (CI) of the survival curve [19]. The choice between the fixed effect model and the random effect model depended on the magnitude of the I^2^ statistic, with the fixed effect model applied for large I^2^ values, and the random effect model for smaller ones. Bayesian network analysis and ranking of the intervention measures by probability were conducted using the gemtc package [20] within R 4.1.0 software. The Markov chain Monte Carlo (MCMC) random/fixed effects model was employed for analysis, with parameters set as follows: initial value set to 2.5, simulation of 4 chains, 5000 annealing steps, and 20000 iterations. The potential scale reduction factor (PSRF) was evaluated, where a value close to 1 (1.00–1.05) indicated good convergence of iterations. Otherwise, increasing the number of simulations and re-evaluating was necessary. Finally, Stata14.0 software was utilized to generate the comparison-correction funnel plot, aiming to identify potential small sample effects and publication bias in the results.

## 3 Results

### 3.1 Literature screening

**Fig. 1:**
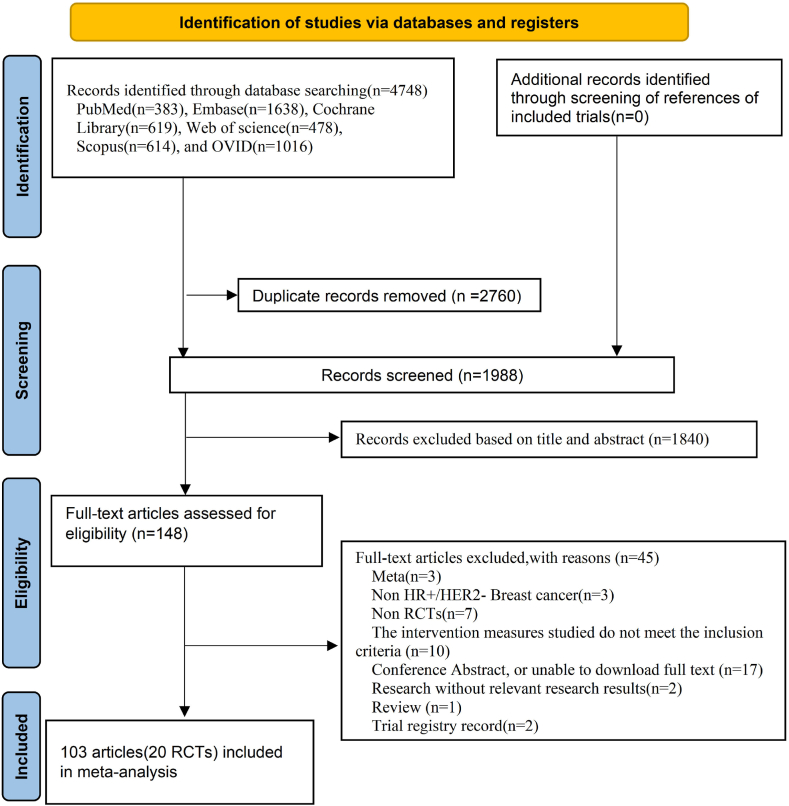
Flowchart of data retrieval and screening.

A total of 4748 articles were initially retrieved from Pubmed (383), Embase (1638), Cochrane Library (619), Web of Science (478), Scopus (614), and OVID (1016). After removing duplicates, 2760 articles were retained. Following the title and abstract screening, 148 literature were selected for full-text review. Ultimately, 103 articles meeting the criteria, comprising 21 RCTs in total, were included for analysis, as illustrated in Fig. 1.

### 3.2 Study characteristics

A total of 20 RCTs were included, comprising 20106 patients. Within these trials, 10772 cases were assigned to the experimental group receiving CDK4/6 inhibitors combined with ET, while 9334 patients were allocated to the control group receiving either CDK4/6 inhibitors or ET alone. The publication dates of included trials spanned from 2014 to 2024, with sample sizes ranging from 53 to 2887 cases. The trials examine four CDK4/6 inhibitors: Abemaciclib (5 studies), Dalpiciclib (2 studies), Palbociclib (8 studies), and Ribociclib (5 studies). The basic characteristics of the included study were provided in Table 1.

**Table 1:** Basic characteristics of the included studies.

ID	Study	Trial identifier	Trial registration	n(man/woman)/Case		Median age/years		Intervention Measures		Period of Treatment/months	Clinical Outcomes
				T	C	T	C	T	C		
1	Rastogi P 2024 [21]	monarchE	NCT03155997	2808(21/2787)	2829(15/2814)	51 (23–89)	51 (22–86)	Abemaciclib + ET	ET	72	③④⑤⑥⑦⑧
2	Slamon DJ 2024 [22]	PALOMA-2	NCT01740427	444(0/444)	222(0/222)	61.7 (10.6)	60.6 (11.2)	Palbociclib + ET	ET	72	③④⑤⑥⑦
3	Kalinsky K 2023 [23]	MAINTAIN Trial	NCT05207709	60(0/60)	59(1/58)	55 (48–67)	59 (51.5–65)	Ribociclib + ET	ET	30	④⑥⑦⑧
4	Slamon DJ 2023 [24]	NATALEE	NCT03701334	334(0/334)	334(0/334)	62 (23–91)	63 (29–88)	Ribociclib + ET	ET	24	①②④⑤⑥⑦⑧
5	Zhang P 2023 [25]	DAWNA-2	NCT03966898	303(0/303)	153(0/153)	54 (47–63)	57 (46–63)	Dalpiciclib + ET	ET	34	①②④⑤⑥
6	Cristofanilli M 2022 [26]	PALOMA-3	NCT01942135	347(0/347)	174(0/174)	56.9 (11.7)	56.8 (10.4)	Palbociclib + ET	ET	72	③④⑤⑥⑦⑧
7	Albanell J 2022 [27]	FLIPPER	NCT02690480	94(0/94)	95(0/95)	64 (38–81)	64 (42–82)	Palbociclib + ET	ET	44	①②④⑤⑥⑦⑧
8	Gnant M 2022 [28]	PALLAS	NCT02513394	2884(17/2867)	2887(19/2868)	52(45–61)	52(45–60)	palbociclib + ET	ET	64	③④⑤⑥⑦⑧
9	Goetz MP 2022 [29]	MONARCH 3	NCT02246621	328(0/328)	165(0/165)	63 (38–87)	63 (32–88)	Abemaciclib + ET	ET	82	①②③④⑤⑥⑦
1010	Hamilton E 2022 [30]	nextMONARCH	NCT02747004	78(0/78)	79(0/79)	53 (32–77)	56 (32–81)	Abemaciclib + ET	Abemaciclib	24	①②③⑧
11	Hortobagyi GN 2022 [31]	MONALEESA-2	NCT01958021	334(0/334)	334(0/334)	61.4 (10.98)	61.9 (10.52)	Ribociclib + ET	ET	69	①②③④⑤⑥⑦
12	Lu YS 2022 [32]	MONALEESA-7	NCT02278120	335(0/335)	337(0/337)	42.6 (6.6)	43.7 (6.17)	Ribociclib + ET	ET	69	①②③④⑤⑥⑦⑧
13	Xu B 2022 [33]	PALOMA-4	NCT02297438	169(0/169)	171(0/171)	54.0 (31,70)	54.0 (29–70)	palbociclib + ET	ET	62	①②③④⑤⑥
14	Loibl S 2021 [34]	PENELOPE-B	NCT01864746	631(0/631)	619(0/619)	49(22,76)	48(19,79)	Palbociclib + ET	ET	60	③④⑤⑥⑦⑧
15	Slamon D2021 [35]	MONALEESA-3	NCT02422615	484(0/484)	242(0/242)	63.4 (9.78)	62.8 (10.59)	Ribociclib + ET	ET	58	①②③④⑤⑥⑦⑧
16	Xu B 2021 [14]	DAWNA-1	NCT03927456	241(0/241)	120(0/120)	50.7 (45.3–59.3)	52.4 (45.5–60.6)	Dalpiciclib + ET	ET	16	①②④⑥
17	Zhang QY 2020 [36] cohort A	MONARCH plus	NCT02763566	207(0/207)	99(0/99)	54(32.0, 83.0)	54(27.0, 77.0)	Abemaciclib + ET	ET	28	①②④⑤⑥⑦⑧
18	Zhang QY 2020 [36] cohort B	MONARCH plus	NCT02763566	104(0/104)	53(0/53)	60(36.0, 80.0)	60(30.0, 80.0)	Abemaciclib + ET	ET	16	①②④⑤⑥⑦⑧
19	Sledge GW 2020 [12]	MONARCH 2	NCT02107703	446(0/446)	223(0/223)	59 (32–91)	62 (32–87)	Abemaciclib + ET	ET	80	①②③④⑤⑥⑦⑧
20	Finn RS 2020 [37]	PALOMA-1	NCT00721409	84(0/84)	81(0/81)	63 (41–89)	64 (38–84)	Palbociclib + ET	ET	55	③④⑤⑦⑧
21	Malorni L 2018 [38]	TREnd trial	NCT02549430	57(0/57)	58(0/58)	67 (37–82)	63 (45–81)	palbociclib + ET	Palbociclib	72	T ②④⑤⑥⑧

T-Experimental group; C-Control group; ①Objective response rate (ORR); ②Disease control rate (DCR); ③Overall survival (OS); ④Progression free survival (PFS); ⑤Adverse reaction events (ARE); ⑥AREs of neutropenia; ⑦AREs of diarrhea; ⑧AREs of fatigue.

### 3.3 Quality assessment

**Fig. 2:**
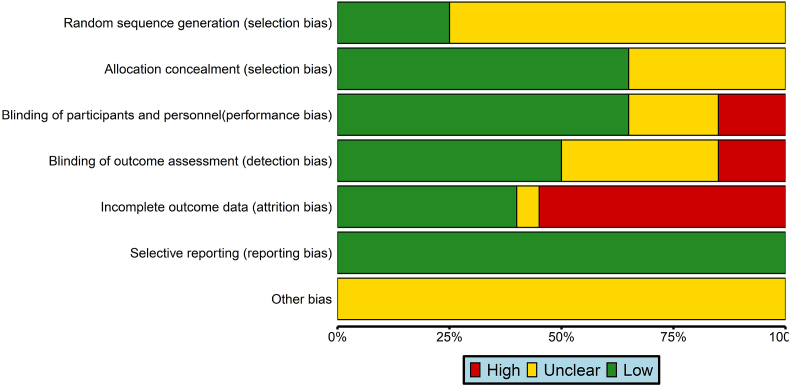
Risk of bias for all included studies.

In the 20 included studies [12,14,[22], [21], [25], [23], [33], [32], [31], [30], [29], [28], [26], [27], [35], [34], [36], [37], [38], [24]], randomization procedures were mentioned, with 5 studies [14,21,25,30,36] employing specific methods for grouping, and hence were rated as having “low risk of bias”. However, other studies [12,22,[23], [31], [32], [33],[26], [27], [28], [29], [34], [35],[24], [37], [38]] did not specify the method of random allocation, resulting in an assessment of “unclear bias risk”. Among them, 13 studies [12,14,22,25,32,31,29,[26], [27], [35],[36], [37], [38]] utilized the masking method for sequence concealment, thus were rated as receiving a “low risk of bias” rating, while 7 studies [21,23,33,30,28,34,24] did not indicate whether sequence concealment was performed and were rated as “Unclear risk of bias”. Furthermore, 3 studies [28,37,38] stated that they were open-label or unblinded to subjects and intervenors, resulting in a “high risk of bias” rating. Another 4 studies [22,21,30,34] did not specify whether blinding was implemented and were thus rated as having an “unclear risk of bias”. Conversely, 13 studies [12,14,[23], [25], [31], [32], [33],^29^,[26], [27], [35],^36^,^24^] indicated that the adoption of a double-blind method received a “Low risk of bias” rating. Regarding evaluator blinding, 3 studies [28,37,38] stated that evaluators were aware of treatment allocation, resulting in a “high risk of bias” rating, while 7 studies [22,21,23,33,30,34,24] did not specify if reporting evaluators were blinded and were rated as “unclear risk of bias”. Conversely, 10 studies [12,14,25,32,31,29,[26], [27], [35],^36^] indicated that evaluators were masked to treatment allocation, and thus were rated as having a “low risk of bias”. Regarding loss to follow-up, 11 studies [14,22,21,30,[26], [27], [28],^34^,[24], [37], [38]] were lost to follow-up, resulting in a “high risk of bias” rating, while one study [33] did not specify whether there was a loss to follow-up and were rated as having an “unclear risk of bias”. However, 8 studies [12,25,23,32,31,29,35,36] demonstrated no loss to follow-up situations and were rated as having a “low risk of bias”. All studies [12,14,[22], [21], [25], [23], [33], [32], [31], [30], [29], [28], [26], [27], [35], [34], [36], [37], [38], [24]] were deemed not to have selectively reported outcomes and received a “low risk of bias” rating in this regard. None of the studies mentioned whether there were other biases, resulting in an “unclear risk of bias” rating. These results were integrated into the quality assessment of research methodology, as illustrated in Fig. 2.

### 3.4 Network evidence

**Fig. 3:**
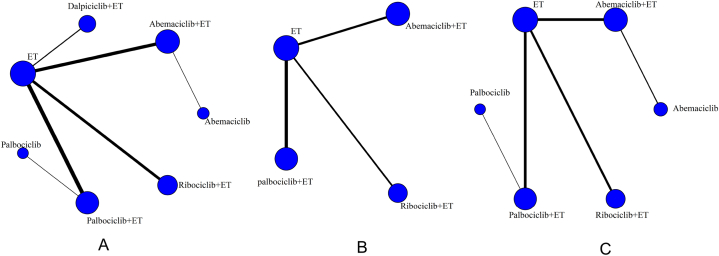
Network maps for of intervention measures. Fig. 3A: ORR, DCR, PFS, overall AREs, fatigue, neutropenia, and diarrhea; Fig. 3B: OS; Fig. 3C: fatigue.

The graph package of R 4.1.0 software was employed to generate the network evidence plots of intervention measures. Fig. 3A illustrated the network evidence plots depicting ORR, DCR, PFS, overall AREs, fatigue, neutropenia, and diarrhea. However, for OS, the data for Dalpiciclib + ET, Abemaciclib, and Palbociclib were not reported in this study, as indicated in Fig. 3B. Furthermore, Fig. 3C showcased the network evidence plots specifically focusing on fatigue, with the fatigue of Dalpiciclib + ET not reported in this study. Given the absence of a closed loop between the studies, only the consistency model was used for statistical analysis.

### 3.5 Outcomes

#### 3.5.1 Efficacy

The network meta-analysis, encompassing 13 studies [12,14,25,[29], [30], [31], [32], [33],^27^,^35^,^36^,^38^,^24^], revealed that combination therapy of Abemaciclib or Ribociclib with ET improved ORR compared to ET alone, with statistical significance (*P* < 0.05). Moreover, Abemaciclib + ET demonstrated a significantly superior ORR compared to Dalpiciclib + ET (*P* < 0.05). A separate analysis of the same 13 studies [12,14,25,[29], [30], [31], [32], [33],^27^,^35^,^36^,^38^,^24^] indicated that the DCR effects of Abemaciclib + ET and Ribociclib + ET were notably stronger than that of ET alone, with statistically significant results (*P* < 0.05). Furthermore, the network meta-analysis results involving 13 studies [12,22,21,[26], [28], [29], [30], [31], [32], [33],^35^,^34^,^37^] demonstrated that Ribociclib + ET can significantly improve the OS of patients with HR+/HER2-breast cancer, exhibiting statistical differences compared with ET alone (*P* < 0.05). In addition, Abemaciclib + ET also displayed improved OS compared to Abemaciclib alone (*P* < 0.05). Lastly, the network meta-analysis involving 20 studies [12,14,[22], [21], [25], [23], [33], [32], [31], [30], [29], [28], [26], [27], [35], [34], [36], [37], [38], [24]] revealed significant improvements in PFS with Abemaciclib + ET, Dalpiciclib + ET, Palbociclib + ET, and Ribociclib + ET improved PFS significantly compared to ET alone (*P* < 0.05). Dalpiciclib + ET improved PFS significantly compared to Abemaciclib + ET, Palbociclib + ET, Ribociclib + ET, Abemaciclib, and Palbociclib (Table 2).

**Table 2:** Network meta-analysis results of clinical efficacy.

Intervention Measures	Abemaciclib + ET	Dalpiciclib + ET	Palbociclib + ET	Ribociclib + ET	Abemacicli	Palbociclib	ET
ORR							
Abemaciclib + ET	0						
Dalpiciclib + ET	1.48 (1.00, 2.38)	0					
Palbociclib + ET	1.37 (0.92, 2.24)	0.93 (0.57, 1.52)	0				
Ribociclib + ET	1.26 (0.95, 1.89)	0.86 (0.58, 1.3)	0.92 (0.61, 1.41)	0			
Abemaciclib	1.44 (0.78, 2.72)	0.97 (0.45, 2.05)	1.05 (0.48, 2.20)	1.13 (0.54, 2.26)	0		
Palbociclib	2.24 (0.62, 9.80)	1.49 (0.40, 6.63)	1.61 (0.47, 6.59)	1.74 (0.48, 7.49)	1.55 (0.37, 7.44)	0	
ET	1.84 (1.49, 2.56)	1.25 (0.91, 1.78)	1.35 (0.95, 1.94)	1.46 (1.17, 1.83)	1.29 (0.66, 2.62)	0.84 (0.20, 3.02)	0
DCR							
Abemaciclib + ET	0						
Dalpiciclib + ET	1.04 (0.91, 1.21)	0					
Palbociclib + ET	1.07 (0.9, 1.26)	1.02 (0.84, 1.23)	0				
Ribociclib + ET	0.97 (0.86, 1.11)	0.93 (0.8, 1.08)	0.91 (0.77, 1.09)	0			
Abemaciclib	1.14 (0.91, 1.45)	1.1 (0.83, 1.44)	1.07 (0.81, 1.44)	1.18 (0.9, 1.53)	0		
Palbociclib	1.27 (0.91, 1.79)	1.22 (0.86, 1.73)	1.19 (0.89, 1.61)	1.31 (0.93, 1.85)	1.11 (0.74, 1.69)	0	
ET	1.1 (1.02, 1.22)	1.06 (0.95, 1.20)	1.04 (0.90, 1.22)	1.14 (1.04, 1.25)	0.97 (0.75, 1.24)	0.87 (0.63, 1.21)	0
OS							
Abemaciclib + ET	0						
Palbociclib + ET	1.11 (0.96, 1.29)		0				
Ribociclib + ET	0.89 (0.76, 1.04)	–	0.8 (0.68, 0.94)				
Abemaciclib	1.61 (1.03, 2.51)	–	1.45 (0.91, 2.31)	1.81(1.13, 2.9)			
ET	1.11 (0.96, 1.29)	–	0.8 (0.68, 0.94)	1.81(1.13, 2.9)	0.74 (0.47,1.16)	–	0
PFS							
Abemaciclib + ET	0						
Dalpiciclib + ET	0.77 (0.61, 0.98)	0					
Palbociclib + ET	1.2 (1.05, 1.37)	1.56 (1.23, 1.97)	0				
Ribociclib + ET	1.04 (0.91, 1.2)	1.35 (1.06, 1.72)	0.87 (0.76, 0.99)	0			
Abemaciclib	1.24 (0.85, 1.82)	1.61 (1.03, 2.51)	1.03 (0.69, 1.55)	1.19 (0.79, 1.79)	0		
Palbociclib	1.74 (1.03, 2.93)	2.26 (1.29, 3.94)	1.45 (0.87, 2.4)	1.67 (0.99, 2.82)	1.4 (0.74, 2.66)	0	
ET	1.66 (1.51, 1.82)	2.15 (1.73, 2.67)	1.38 (1.26, 1.51)	1.59 (1.43, 1.76)	1.34 (0.9, 1.97)	0.95 (0.57, 1.59)	0

#### 3.5.2 AREs

AREs were statistically analyzed across 18 studies [12,[21], [22], [23], [25], [31], [32], [33],[24], [26], [27], [28], [29], [34], [35], [36], [37], [38]]. The results of network meta-analysis indicated that when combined with ET, Abemaciclib, Dalpiciclib, Palbociclib, and Ribociclib led to significantly higher rates of AREs compared to ET alone (*P* < 0.05). Specially, Dalpiciclib + ET showed a notably higher incidence of AREs compared to Ribociclib + ET. In 19 studies [12,14,[21], [22], [23], [25], [31], [32], [33],[24], [26], [27], [28], [29], [34], [35], [36], [37], [38]], statistical analysis was performed on the incidence of neutropenia. The results revealed that compared to ET alone, Abemaciclib, Dalpiciclib, Palbociclib, and Ribociclib combined with ET significantly elevated the incidence rate of leukopenia (*P* < 0.05). Notably, the incidence of neutropenia induced by Palbociclib was significantly higher compared to ET alone. Furthermore, Dalpiciclib + ET demonstrated a propensity to induce neutropenia in patients, with its incidence significantly surpassing that of Palbociclib + ET, Ribociclib + ET, Abemaciclib alone, and Palbociclib alone, thus suggesting that Dalpiciclib might be the primary culprit behind neutropenia. Examining 16 studies [12,22,21,23,[26], [27], [28], [29], [30], [31], [32], [34], [35], [36], [37],^24^] focused on the incidence of diarrhea, the network meta-analysis results displayed that Abemaciclib + ET and Abemaciclib alone triggered significantly higher rates of diarrhea compared to ET alone, demonstrating statistical significance (*P* < 0.05). Moreover, Abemaciclib + ET induced markedly higher rates of diarrhea compared to Palbociclib + ET and Ribociclib + ET (*P* < 0.05). A thorough analysis of fatigue across 14 studies [12,21,23,32,30,[24], [26], [27], [28], [34], [35], [36], [37], [38]] revealed that Abemaciclib + ET, Palbociclib + ET, and Palbociclib alone were associated with a significant increase in the incidence of fatigue (*P* < 0.05) (Table 3).

**Table 3:** Network meta-analysis results of AREs.

Variables	Abemaciclib + ET	Dalpiciclib + ET	Palbociclib + ET	Ribociclib + ET	Abemacicli	Palbociclib	ET
ARE							
Abemaciclib + ET	0						
Dalpiciclib + ET	0.31 (0.09, 1.05)	0					
Palbociclib + ET	0.81 (0.43, 1.53)	2.62 (0.80, 8.54)	0				
Ribociclib + ET	1.16 (0.57, 2.43)	3.78(1.1,13.16)	1.44 (0.74, 2.85)	0			
Abemaciclib	1.03 (0.35, 3.01)	3.34(0.66,16.7)	1.28 (0.37, 4.43)	0.88 (0.24, 3.21)	0		
Palbociclib	0.86 (0.25, 2.98)	2.81(0.56,13.78)	1.07 (0.37, 3.09)	0.74 (0.21, 2.59)	0.84 (0.16, 4.31)	0	
ET	2.19 (1.36, 3.59)	7.1 (2.32, 21.58)	2.71 (1.80, 4.09)	1.88 (1.10, 3.21)	2.13 (0.66, 6.89)	2.53 (0.82, 7.86)	0
Neutropenia							
Abemaciclib + ET	0						
Dalpiciclib + ET	0.00 (0.00, 0.00)	0					
Palbociclib + ET	0.08 (0.01, 0.66)	6.24 e+18 (37.2, 1.19e+64)	0				
Ribociclib + ET	0.17 (0.01, 2.05)	1.30e+19 (77.48, 3.15e+64)	2.17 (0.19, 22.14)	0			
Abemaciclib	0.76 (0.04, 14.11)	6.12e+19 472 (308.73, 1.18e+65)	9.72 (0.26, 330.33)	4.49 (0.09, 203.50)	0		
Palbociclib	0.08 (0.00, 3.03)	6.57e+18 (32.61, 1.35e+64)	1.07 (0.06, 18.54)	0.50 (0.01, 21.43)	0.11 (0.00, 10.81)	0	
ET	6.61 (1.57, 38.63)	5.47e+20 (3578.16, 1.045e+66)	84.17 (22.78, 379.73)	38.81 (6.50, 318.6)	8.66 (0.37, 279.24)	79.12 (3.54, 2182.75)	0
Diarrhea							
Abemaciclib + ET	0						
Palbociclib + ET	24.14 (2.74, 361.92)	–	0				
Ribociclib + ET	21.28 (1.66, 327.87)	–	0.88 (0.07, 7.67)	0			
Abemaciclib	0.26 (0.00, 8.13)	–	0.01 (0, 0.59)	0.01 (0.00, 0.85)			
ET	32.53 (5.85, 298.47)	–	1.36 (0.31, 5.50)	1.54 (0.28, 10.66)	132.57 (2.88, 16122.36)		0
Fatigue							
Abemaciclib + ET	0						
Palbociclib + ET	3.95 (0.23, 40.16)	–	0				
Ribociclib + ET	2.08 (0.05, 33.01)	–	0.54 (0.02, 6.2)	0			
Abemaciclib	1.61 (0.18, 16.24)	–	0.41 (0.02, 17.13)	0.78 (0.02, 70.57)	0		
Palbociclib	0 (0.00, 2.18)	–	0.00 (0.00, 0.5)	0.00 (0.00, 1.23)	0.00 (0.00, 1.60)	0	
ET	13.46 (1.67, 117.4)	–	3.38 (1.08, 19.45)	6.41 (0.94, 132.01)	8.4 (0.37, 179.54)	320789798140.86 (6.79, 9.85e+32)	0

#### 3.5.3 Intervention ranking

The gemtc package of R 4.1.0 software was employed to conduct a Bayesian network meta-analysis, aiming to evaluate the efficacy and safety of each intervention across various indicators. The outcomes revealed that the following rankings based on their efficacy in improving patients’ ORR, DCR, OS time and PFS time in HR+/HER2-breast canceer patients, as well as the incidence of AREs, neutropenia, diarrhea, and fatigue: (1) improvement of ORR (from high to low): Abemaciclib + ET > Ribociclib + ET > Palbociclib + ET > Abemaciclib > Dalpiciclib + ET > Palbociclib > ET; (2) improvement of DCR (from high to low): Ribociclib + ET > Abemaciclib + ET > Dalpiciclib + ET > Palbociclib + ET > ET > Abemaciclib > Palbociclib; (3) prolongation of OS time in patients with HR+/HER2-breast cancer (from high to low): Ribociclib + ET > Abemaciclib + ET > Palbociclib + ET > ET > Abemaciclib; (4) prolongation of PFS time in patients with HR+/HER2-breast cancer (from high to low): Dalpiciclib + ET > Abemaciclib + ET > Ribociclib + ET > Abemaciclib > Palbociclib + ET > Palbociclib > ET; (5) incidence of AREs ranking (from high to low): Dalpiciclib + ET > Palbociclib + ET > Palbociclib > Abemaciclib + ET > Abemaciclib > Ribociclib + ET > ET; (6) incidence of neutropenia (high to low): Dalpiciclib + ET > Palbociclib + ET > Palbociclib > Ribociclib + ET > Abemaciclib > Abemaciclib + ET > ET; (7) incidence of diarrhea (high to low): Abemaciclib > Abemaciclib + ET > Ribociclib + ET > Palbociclib + ET > ET; (8) incidence of fatigue (high to low): Palbociclib > Abemaciclib + ET > Abemaciclib > Ribociclib + ET > Palbociclib + ET > ET (Table 4).

**Table 4:** Rank sorting results of intervention efficacy.

Intervention Measures	ORR		DCR		OS		PFS		ARE	
	Probability	Rank	Probability	Rank	Probability	Rank	Probability	Rank	Probability	Rank
Abemaciclib + ET	0.94	1	0.77	2	0.74	2	0.76	2	0.52	4
Dalpiciclib + ET	0.45	5	0.59	3	–	–	0.99	1	0.04	7
Palbociclib + ET	0.55	3	0.5	4	0.47	3	0.42	5	0.35	6
Ribociclib + ET	0.66	2	0.88	1	0.98	1	0.67	3	0.64	2
Abemaciclib	0.49	4	0.3	6	0.04	5	0.43	4	0.54	3
Palbociclib	0.25	6	0.15	7	–	–	0.12	6	0.44	5
ET	0.16	7	0.31	5	0.26	4	0.11	7	0.97	1

Intervention Measures	ARE of Neutropenia		ARE of Diarrhea		ARE of Fatigue	
	Probability	Rank	Probability	Rank	Probability	Rank
Abemaciclib + ET	0.74	2	0.2	4	0.35	5
Dalpiciclib + ET	0	7	–	–	–	–
Palbociclib + ET	0.3	6	0.71	2	0.66	2
Ribociclib + ET	0.45	4	0.68	3	0.51	3
Abemaciclib	0.68	3	0.06	5	0.49	4
Palbociclib	0.35	5	–	–	0.02	6
ET	0.98	1	0.85	1	0.97	1

### 3.6 Publication bias assessment

**Fig. 4:**
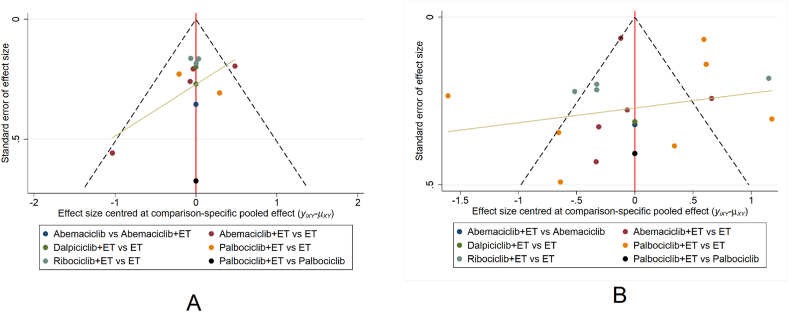
Comparison-correction funnel diagram. Fig. 4A: ORR; Fig. 4B: AREs. **Note:** Dots symbolize various included studies, while different colors signify distinct interventions.

This study underwent an assessment for publication bias and small sample effects, focusing on the primary indicator, ORR, and the secondary indicator, AREs. Stata 14.0 was used to generate a comparison-corrected funnel plot. The funnel plots for ORR and AREs demonstrate a symmetrical distribution of all studies around the vertical line at x = 0. This symmetry suggested a decreased likelihood of significant publication bias. Furthermore, all data points fall within the confines of the triangle, indicating the absence of small sample effects in both ORR (Fig. 4A) and AREs (Fig. 4B).

## 4 Discussion

In this study, we employed a network meta-analysis to evaluate the efficacy (ORR, DCR, OS, and PFS) and safety (total AREs, neutropenia, diarrhea, and fatigue) of CDK4/6 inhibitors combined with ET in HR+/HER2-breast cancer. The findings from indirect comparisons drawn from 20 RCTs showed that CDK4/6 inhibitors combined with ET exhibit superior efficacy over standalone ET and CDK4/6 inhibitors alone, both in the short-term and long-term efficacy assessments. Furthermore, the clinical efficacy observed for Abemaciclib, Dalpiciclib, Ribociclib, and Palbociclib combined with ET was found to be comparable in terms of ORR, DCR, and PFS, consistent with previous research findings [8].

In terms of short-term efficacy, CDK4/6 inhibitors combined with ET have demonstrated notable improvements in patients' ORR and DCR, showcasing statistically significant differences compared to ET alone. For instance, a study highlighted significant enhancements in ORR and PFS with the combination of Abemaciclib and a non-steroidal aromatase inhibitor as evidenced by the interim analysis of MONARCH 3 [39]. In addition, Lyu [40] conducted a retrospective analysis focusing on patients with HR+/HER2-breast cancer treated with Palbociclib + ET. The findings revealed a short-term ORR of 48.8 % and a DCR of 88.4 %, with a median PFS of 12 months. Moreover, the ORR of Ribociclib combined with fulvestrant in postmenopausal patients with HR+/HER2-breast cancer as first-line agents has been significantly enhanced [41].

In terms of OS, Ribociclib + ET demonstrated a significant improvement compared to Palbociclib + ET, Abemaciclib, and ET. Abemaciclib + ET also exhibited an OS extension, with a statistically significant difference compared to Abemaciclib alone. A phase Ш clinical trial revealed that Ribociclib + ET, with a median OS not yet reached, significantly surpassed placebo + ET with a median OS of 40.9 months (HR 0.712, 95 % CI 0.54–0.95, *P* < 0.00973) [42]. Another phrase Ш study [42] corroborated the findings, demonstrating significant OS extension with ribociclib combined with fulvestrant in postmenopausal breast cancer patients. In MONARCH 2, Abemaciclib + fulvestrant displayed a median OS of 46.7 months, contrasting with 37.3 months for placebo + fulvestrant (HR 0.757; 95 % CI 0.606–0.945, *P* = 0.01) [12]. Furthermore, CDK4/6 inhibitors combined with ET exhibited improved PFS compared to ET alone. Dalpiciclib + ET notably enhanced PFS versus Abemaciclib + ET, Dalpiciclib + ET, Palbociclib + ET and Ribociclib + ET, Abemaciclib, and Palbociclib, while Ribociclib + ET outperformed Palbociclib + ET, which is consistent with the cross-trial matching-adjusted indirect comparison conducted by Jhaveri et al. [43]. Network meta-analysis indicated that Dalpiciclib + fulvestrant is the most effective combination for extending PFS, supported by a surface under the cumulative ranking curve (SUCRA) of 85.0 % [44]. These findings validate the efficacy of CD4/6 inhibitors combined with ET in improving os and PFS in HR+/HER2-breast cancer patients.

Although these Four drugs exhibit similar mechanisms of action and therapeutic effects in terms of ORR and DCR, discrepancies exist in their safety profiles. Our analysis revealed that Dalpiciclib + ET in the treatment of HR+/HER2-breast cancer led to the highest incidence of grade 3/4 AREs, such as neutropenia. Regarding diarrhea as an ARE, Abemaciclib + ET exhibited a higher incidence compared to Palbociclib + ET and Ribociclib + ET, which may be linked to alterations of gut microbiota signatures [45]. The DAWNA-1 study further confirmed that the most common grade 3 or 4 AREs were neutropenia (84.2 %) and leukopenia (62.1 %) [14]. Dalpiciclib + ET was potentially the most effective combination for extending PFS but demonstrated increased toxicity and failed to achieve an OS advantage. Dalpiciclib's broader inhibitory effects, including highly selective inhibition of CDK4/6, may result in stronger neutrophil inhibition and subsequent neutropenia side effects [46]. Common AREs associated with Abemaciclib + ET in advanced breast cancer treatment included diarrhea and nausea [13]. Abemaciclib exhibits a distinct safety profile with higher gastrointestinal toxicity, possibly due to its greater potency against CDK4 than CDK6 and additional potency against CDK9 [47,48].

Limitations of this study include: (1) some studies did not specify allocation methods; (2) small sample size and limited RCT for Abemaciclib and Palbociclib alone; (3) lack of comparative studies on Dalpiciclib and Ribociclib alone, potentially impacting the generalizability of findings; (4) inability to observe inconsistencies due to the absence of a closed loop in the network graph. (5) insufficient data on OS information for Dalpiciclib, Dalpiciclib + ET and Palbociclib. Therefore, further studies are warranted to evaluate the OS advantage of Dalpiciclib and Palbociclib when utilized in first-line endocrine treatment.

## 5 Conclusion

In summary, the clinical efficacy in terms of ORR and DCR observed with Abemaciclib + ET, Dalpiciclib + ET, Palbociclib + ET, and Ribociclib + ET is comparable and superior to that of ET alone. Ribociclib + ET exhibited significant improvements in both PFS and OS compared to ET alone. Dalpiciclib + ET notably enhanced PFS compared to Abemaciclib + ET, Dalpiciclib + ET, Palbociclib + ET, Ribociclib + ET, Abemaciclib alone, and Palbociclib alone. However, concerning AREs, Dalpiciclib + ET was associated with the highest incidence of grade 3/4 AREs, particularly neutropenia. Abemaciclib + ET exhibited a significantly higher incidence of diarrhea compared to Palbociclib + ET and Ribociclib + ET. Due to the absence of direct comparisons between certain drugs, the efficacy and safety conclusions regarding CDK4/6 inhibitors combined with ET in this study warrant further confirmation through rigorous, scientific, large-sample, and high-quality clinical studies.

## Data availability

Data will be made available on request.

## Funding statement

Not applicable.

## Ethics approval and consent to participate

Not applicable.

## CRediT authorship contribution statement

**Fei Tong:** Writing – original draft, Methodology, Formal analysis, Data curation. **Yi Lu:** Writing – review & editing, Writing – original draft, Project administration, Formal analysis, Conceptualization. **Hong-Fang Ma:** Writing – review & editing, Formal analysis. **Jun Shen:** Writing – review & editing, Supervision, Investigation, Conceptualization.

## Declaration of competing interest

The authors declare that they have no known competing financial interests or personal relationships that could have appeared to influence the work reported in this paper.
