# Supplementary material for: Comparative efficacy and safety of CDK4/6 inhibitors combined with endocrine therapies for HR+/HER2-breast cancer: Systematic review and network meta-analysis
Source: Heliyon. 2024 May 21;10(11):e31583. doi: 10.1016/j.heliyon.2024.e31583 (PMC11145204; doi:10.1016/j.heliyon.2024.e31583)
Supplement: Multimedia component 1 [file mmc1.docx]

1. PubMed

|  | **Search Strategy** | **Results** |
| --- | --- | --- |
|  | (#1 AND #2 AND #3 AND #4 AND #5) | 383 |
| #1  #2  #3  #4  #5  #6 | ((((((((((((("breast cancer*"[Title/Abstract]) OR ("breast malignancy"[Title/Abstract])) OR ("Breast Malignant Tumor*"[Title/Abstract])) OR ("Breast Neoplasm*"[Title/Abstract])) OR ("Breast Tumor*"[Title/Abstract])) OR ("Breast Carcinoma*"[Title/Abstract])) OR ("Cancer of Breast*"[Title/Abstract])) OR ("Mammary Carcinoma*"[Title/Abstract])) OR ("Mammary Cancer*"[Title/Abstract])) OR ("Mammary Neoplasm*"[Title/Abstract])) OR ("breast malignan*"[Title/Abstract])) OR ("Malignant Tumor of Breast"[Title/Abstract])) OR ("Mammary Cancer*"[Title/Abstract])) OR ("Malignant Tumor of Breast*"[Title/Abstract])  ((((((((("Hormone Receptor positive"[Title/Abstract]) OR ("HR-positive"[Title/Abstract])) OR ("HR+"[Title/Abstract])) OR ("HR positive"[Title/Abstract])) OR ("Hormone Receptor-positive"[Title/Abstract])) OR ("human epidermal growth factor receptor 2 negative"[Title/Abstract])) OR ("HER2-negative"[Title/Abstract])) OR ("HER2-"[Title/Abstract])) OR ("HER2 negative"[Title/Abstract])) OR ("human epidermal growth factor receptor 2-negative"[Title/Abstract])  (((((((((((((("endocrine therapy"[Title/Abstract]) OR ("tamoxifen"[Title/Abstract])) OR ("aromatase inhibitor"[Title/Abstract])) OR ("Antineoplastic Agents, Hormonal"[Title/Abstract])) OR ("Aromatase Inhibitor*"[Title/Abstract])) OR ("Selective Estrogen Receptor Modulator*"[Title/Abstract])) OR ("Tamoxifen"[Title/Abstract])) OR ("ICI-46474"[Title/Abstract])) OR ("ICI-47699"[Title/Abstract])) OR ("Nolvadex"[Title/Abstract])) OR ("Novaldex"[Title/Abstract])) OR ("Soltamox"[Title/Abstract])) OR ("Tamoxifen Citrate"[Title/Abstract])) OR ("Zitazonium"[Title/Abstract]) OR ("aromatase inhibitor*"[Title/Abstract])  ((((((("CDK4/6 inhibitor*"[Title/Abstract]) OR ("CDK 4 6 inhibitor*"[Title/Abstract])) OR ("cyclin-dependent kinase 4 6 inhibitor*"[Title/Abstract])) OR ("palbociclib"[Title/Abstract])) OR ("ribociclib"[Title/Abstract])) OR ("abemaciclib"[Title/Abstract])) OR ("Trilaciclib"[Title/Abstract])) OR ("dalpiciclib"[Title/Abstract])  (((((((((("Randomized controlled trial"[Title/Abstract]) OR ("controlled clinical trial"[Title/Abstract])) OR ("random allocation"[Title/Abstract])) OR ("double-blind"[Title/Abstract])) OR ("single-blind"[Title/Abstract])) OR ("randomly"[Title/Abstract])) OR ("randomized"[Title/Abstract])) OR ("clinical trial"[Title/Abstract])) OR ("trial"[Title/Abstract])) OR ("RCT*"[Title/Abstract])) OR ("random"[Title/Abstract])  #1 AND #2 AND #3 AND #4 AND #5 |  |

2. Embase

|  | Search Strategy | Results |
| --- | --- | --- |
|  | (#1 AND #2 AND #3 AND #4 AND #5) | 1638 |
| #1  #2  #3  #4  #5  #6  #7  #8  #9  #9  #10  #11  #12 | 'breast tumor'/exp  'breast cancer*':ab,ti OR 'breast malignancy':ab,ti OR 'breast malignant tumor*':ab,ti OR 'breast neoplasm*':ab,ti OR 'breast tumor*':ab,ti OR 'breast carcinoma*':ab,ti OR 'cancer of breast*':ab,ti OR 'mammary carcinoma*':ab,ti OR 'mammary neoplasm*':ab,ti OR 'breast malignan*':ab,ti OR 'malignant tumor of breast':ab,ti OR 'mammary cancer*':ab,ti OR 'malignant tumor of breast*':ab,ti  'hormone receptor positive':ab,ti OR 'hr-positive':ab,ti OR 'hr+':ab,ti OR 'hr positive':ab,ti OR 'hormone receptor-positive':ab,ti  'human epidermal growth factor receptor 2 negative':ab,ti OR 'her2-negative':ab,ti OR 'her2-':ab,ti OR 'her2 negative':ab,ti OR 'human epidermal growth factor receptor 2-negative':ab,ti  'endocrine therapy'/exp  'endocrine therapy':ab,ti OR 'aromatase inhibitor':ab,ti OR 'antineoplastic agents, hormonal':ab,ti OR 'selective estrogen receptor modulator*':ab,ti OR 'tamoxifen':ab,ti OR 'ici-46474':ab,ti OR 'ici-47699':ab,ti OR 'nolvadex':ab,ti OR 'novaldex':ab,ti OR 'soltamox':ab,ti OR 'tamoxifen citrate':ab,ti OR 'tomaxithen':ab,ti OR 'zitazonium':ab,ti OR 'aromatase inhibitor*':ab,ti  'cdk4/6 inhibitor*':ab,ti OR 'cdk 4 6 inhibitor*':ab,ti OR 'cyclin-dependent kinase 4 6 inhibitor*':ab,ti OR 'palbociclib':ab,ti OR 'ribociclib':ab,ti OR 'abemaciclib':ab,ti OR 'trilaciclib':ab,ti OR 'dalpiciclib':ab,ti  'controlled study'/exp  'randomize control trial' OR (randomize AND ('control'/exp OR control) AND ('trial'/exp OR trial)) OR 'controlled clinical trial'/exp OR 'controlled clinical trial' OR (controlled AND ('clinical'/exp OR clinical) AND ('trial'/exp OR trial)) OR 'random allocation'/exp OR 'random allocation' OR (random AND allocation) OR 'double blind' OR 'single blind' OR randomly OR randomized OR 'clinical trial'/exp OR 'clinical trial' OR (('clinical'/exp OR clinical) AND ('trial'/exp OR trial)) OR 'trial'/exp OR trial OR rct OR random  #1 OR #2  #5 OR #6  #8 OR #9  #3 AND #4 AND #10 AND #11 AND #12 |  |

3. Cochrane Library

|  | **Search Strategy** | **Results** |
| --- | --- | --- |
|  | (#1 AND #2 AND #3) | 619 |
| #1  #2  #3  #4  #5  #6  #7  #8  #9  #10  #11 | MeSH descriptor: [Breast Neoplasms] explode all trees  ("breast cancer" OR "breast malignancy" OR "Breast Malignant Tumor" OR "Breast Neoplasm" OR "Breast Tumor" OR "Breast Carcinoma" OR "Cancer of Breast" OR "Mammary Carcinoma" OR "Mammary Cancer" OR "Mammary Neoplasm" OR "breast malignan" OR "Malignant Tumor of Breast" OR "Mammary Cancer" OR "Malignant Tumor of Breast" OR "breast cancers" OR "Breast Malignant Tumors" OR "Breast Neoplasms" OR "Breast Tumors" OR "Breast Carcinomas" OR "Cancer of Breasts" OR "Mammary Carcinomas" OR "Mammary Cancers" OR "Mammary Neoplasms" OR "breast malignans" OR "Mammary Cancers" OR "Malignant Tumor of Breasts"):ti,ab,kw  ("Hormone Receptor positive" OR "HR-positive" OR "HR+" OR "HR positive" OR "Hormone Receptor-positive"):ti,ab,kw  ("human epidermal growth factor receptor 2 negative" OR "HER2-negative" OR "HER2-" OR "HER2 negative" OR "human epidermal growth factor receptor 2-negative"):ti,ab,kw  ("endocrine therapy" OR "tamoxifen" OR "aromatase inhibitor" OR "Antineoplastic Agents, Hormonal" OR "Aromatase Inhibitor" OR "Selective Estrogen Receptor Modulator" OR "Tamoxifen" OR "ICI-46474" OR "ICI-47699" OR "Nolvadex" OR "Novaldex" OR "Soltamox" OR "Tamoxifen Citrate" OR "Tomaxithen" OR "Zitazonium" OR "aromatase inhibitor" OR "Aromatase Inhibitors" OR "Selective Estrogen Receptor Modulators" OR "aromatase inhibitors"):ti,ab,kw  ("CDK4/6 inhibitor" OR "CDK 4 6 inhibitor" OR "cyclin-dependent kinase 4 6 inhibitor" OR "palbociclib" OR "dalpiciclib" OR "ribociclib" OR "abemaciclib" OR "Trilaciclib" OR "CDK4/6 inhibitors" OR "CDK 4 6 inhibitors" OR "cyclin-dependent kinase 4 6 inhibitors"):ti,ab,kw  MeSH descriptor: [Randomized Controlled Trial] explode all trees  ("Randomized controlled trial" OR "controlled clinical trial" OR "random allocation" OR "double-blind" OR "single-blind" OR "randomly" OR "randomized" OR "clinical trial" OR "trial" OR "RCT" OR "random" OR "RCTs"):ti,ab,kw  #1 OR #2  #7 OR #8  #9 AND #3 AND #4 AND #5 AND #6 AND #10 |  |

4. Web of Science

|  | **Search Strategy** | **Results** |
| --- | --- | --- |
|  | #1 AND #2 AND #3 | 478 |
| #1  #2  #3  #3  #4  #5  #6 | (TS=("breast cancer*" OR "breast malignancy" OR "Breast Malignant Tumor*" OR "Breast Neoplasm*" OR "Breast Tumor*" OR "Breast Carcinoma*" OR "Cancer of Breast*" OR "Mammary Carcinoma*" OR "Mammary Cancer*" OR "Mammary Neoplasm*" OR "breast malignan*" OR "Malignant Tumor of Breast" OR "Mammary Cancer*" OR "Malignant Tumor of Breast*")) OR AB=("breast cancer*" OR "breast malignancy" OR "Breast Malignant Tumor*" OR "Breast Neoplasm*" OR "Breast Tumor*" OR "Breast Carcinoma*" OR "Cancer of Breast*" OR "Mammary Carcinoma*" OR "Mammary Cancer*" OR "Mammary Neoplasm*" OR "breast malignan*" OR "Malignant Tumor of Breast" OR "Mammary Cancer*" OR "Malignant Tumor of Breast*") OR AB=("breast cancer*" OR "breast malignancy" OR "Breast Malignant Tumor*" OR "Breast Neoplasm*" OR "Breast Tumor*" OR "Breast Carcinoma*" OR "Cancer of Breast*" OR "Mammary Carcinoma*" OR "Mammary Cancer*" OR "Mammary Neoplasm*" OR "breast malignan*" OR "Malignant Tumor of Breast" OR "Mammary Cancer*" OR "Malignant Tumor of Breast*")  (TS=("Hormone Receptor positive" OR "HR-positive" OR "HR+" OR "HR positive" OR "Hormone Receptor-positive" )) OR AB=("Hormone Receptor positive" OR "HR-positive" OR "HR+" OR "HR positive" OR "Hormone Receptor-positive")  (TS=("human epidermal growth factor receptor 2 negative" OR "HER2-negative" OR "HER2-" OR "HER2 negative" OR "human epidermal growth factor receptor 2-negative")) OR AB=("human epidermal growth factor receptor 2 negative" OR "HER2-negative" OR "HER2-" OR "HER2 negative" OR "human epidermal growth factor receptor 2-negative")  (TS=("endocrine therapy" OR "tamoxifen" OR "aromatase inhibitor" OR "Antineoplastic Agents, Hormonal" OR "Aromatase Inhibitor*" OR "Selective Estrogen Receptor Modulator*" OR "Tamoxifen" OR "ICI-46474" OR "ICI-47699" OR "Nolvadex" OR "Novaldex" OR "Soltamox" OR "Tamoxifen Citrate" OR "Tomaxithen" OR "Zitazonium" OR "aromatase inhibitor*")) OR AB=("endocrine therapy" OR "tamoxifen" OR "aromatase inhibitor" OR "Antineoplastic Agents, Hormonal" OR "Aromatase Inhibitor*" OR "Selective Estrogen Receptor Modulator*" OR "Tamoxifen" OR "ICI-46474" OR "ICI-47699" OR "Nolvadex" OR "Novaldex" OR "Soltamox" OR "Tamoxifen Citrate" OR "Tomaxithen" OR "Zitazonium" OR "aromatase inhibitor*")  (TS=("CDK4/6 inhibitor*" OR "CDK 4 6 inhibitor*" OR "cyclin-dependent kinase 4 6 inhibitor*" OR "palbociclib" OR "ribociclib" OR "abemaciclib" OR "dalpiciclib" OR "Trilaciclib")) OR AB=("CDK4/6 inhibitor*" OR "CDK 4 6 inhibitor*" OR "cyclin-dependent kinase 4 6 inhibitor*" OR "palbociclib" OR "ribociclib" OR "abemaciclib" OR "dalpiciclib" OR "Trilaciclib")  (TS=("Randomized controlled trial" OR "controlled clinical trial" OR "random allocation" OR "double-blind" OR "single-blind" OR "randomly" OR "randomized" OR "clinical trial" OR "trial" OR "RCT*" OR "random")) OR AB=("Randomized controlled trial" OR "controlled clinical trial" OR "random allocation" OR "double-blind" OR "single-blind" OR "randomly" OR "randomized" OR "clinical trial" OR "trial" OR "RCT*" OR "random")  #1 AND #2 AND #3 AND #4 AND #5  Índices: SCI-EXPANDED, SSCI, A&HCI, CPCI-S, CPCI-SSH, ESCI. Stipulated time: Every year |  |

5. Scopus

|  | **Search Strategy** | **Results** |
| --- | --- | --- |
|  | #1 AND #2 AND #3 | 614 |
| #1  #2  #3  #4  #5  #6 | TITLE-ABS-KEY("breast cancer*" OR "breast malignancy" OR "Breast Malignant Tumor*" OR "Breast Neoplasm*" OR "Breast Tumor*" OR "Breast Carcinoma*" OR "Cancer of Breast*" OR "Mammary Carcinoma*" OR "Mammary Cancer*" OR "Mammary Neoplasm*" OR "breast malignan*" OR "Malignant Tumor of Breast" OR "Mammary Cancer*" OR "Malignant Tumor of Breast*")  TITLE-ABS-KEY("Hormone Receptor positive" OR "HR-positive" OR "HR+" OR "HR positive" OR "Hormone Receptor-positive")  TITLE-ABS-KEY("human epidermal growth factor receptor 2 negative" OR "HER2-negative" OR "HER2-" OR "HER2 negative" OR "human epidermal growth factor receptor 2-negative")  TITLE-ABS-KEY("endocrine therapy" OR "tamoxifen" OR "aromatase inhibitor" OR "Antineoplastic Agents, Hormonal" OR "Aromatase Inhibitor*" OR "Selective Estrogen Receptor Modulator*" OR "Tamoxifen" OR "ICI-46474" OR "ICI-47699" OR "Nolvadex" OR "Novaldex" OR "Soltamox" OR "Tamoxifen Citrate" OR "Tomaxithen" OR "Zitazonium" OR "aromatase inhibitor*")  TITLE-ABS-KEY("CDK4/6 inhibitor*" OR "CDK 4 6 inhibitor*" OR "cyclin-dependent kinase 4 6 inhibitor*" OR "palbociclib" OR "ribociclib" OR "abemaciclib" OR "Trilaciclib" OR "dalpiciclib")  TITLE-ABS-KEY("Randomized controlled trial" OR "controlled clinical trial" OR "random allocation" OR "double-blind" OR "single-blind" OR "randomly" OR "randomized" OR "clinical trial" OR "trial" OR "RCT*" OR "random")  #1 AND #2 AND #3 AND #4 AND #5 AND #6 |  |

6. OVID

|  | **Search Strategy** | **Results** |
| --- | --- | --- |
| **#** | #1 AND #2 AND #3 | 1012 |
| 1  2  3  4  5  6  7 | ("breast cancer*" or "breast malignancy" or "Breast Malignant Tumor*" or "Breast Neoplasm*" or "Breast Tumor*" or "Breast Carcinoma*" or "Cancer of Breast*" or "Mammary Carcinoma*" or "Mammary Cancer*" or "Mammary Neoplasm*" or "breast malignan*" or "Malignant Tumor of Breast" or "Mammary Cancer*" or "Malignant Tumor of Breast*").ab,kw,ti. ("Hormone Receptor positive" or "HR-positive" or "HR+" or "HR positive" or "Hormone Receptor-positive").ab,kw,ti.  ("human epidermal growth factor receptor 2 negative" or "HER2-negative" or "HER2-" or "HER2 negative" or "human epidermal growth factor receptor 2-negative").ab,kw,ti.  ("endocrine therapy" or "tamoxifen" or "aromatase inhibitor" or "Antineoplastic Agents, Hormonal" or "Aromatase Inhibitor*" or "Selective Estrogen Receptor Modulator*" or "Tamoxifen" or "ICI-46474" or "ICI-47699" or "Nolvadex" or "Novaldex" or "Soltamox" or "Tamoxifen Citrate" or "Tomaxithen" or "Zitazonium" or "aromatase inhibitor*").ab,kw,ti.  ("CDK4/6 inhibitor*" or "CDK 4 6 inhibitor*" or "cyclin-dependent kinase 4 6 inhibitor*" or "palbociclib" or "ribociclib" or "abemaciclib" or "Trilaciclib" or "dalpiciclib").ab,kw,ti.  ("Randomized controlled trial" or "controlled clinical trial" or "random allocation" or "double-blind" or "single-blind" or "randomly" or "randomized" or "clinical trial" or "trial" or "RCT*" or "random").ab,kw,ti.  1 and 2 and 3 and 4 and 5 and 6 |  |
